# Transition from an MBBS to an MD program – Using innovation and thinking outside the square

**DOI:** 10.15694/mep.2019.000197.1

**Published:** 2019-11-04

**Authors:** Janie Dade Smith, Elizabeth Edwards, Peter D Jones, Colleen Cheek, Richard B Hays

**Affiliations:** 1 Bond University; 2 York St John University; 3 University of Tasmania

**Keywords:** Curriculum change, Medical education, Doctor of Medicine.

## Abstract

This article was migrated. The article was marked as recommended.

Background

There has been a trend globally to move from a Bachelor of Medicine, Bachelor of Surgery (MBBS) to a Doctor of Medicine (MD) for primary medical education. This shift has seen many Australian universities change to an MD, mostly from graduate entry programs. This paper describes the novel and unique 3+2 model from one Australian university, that enabled undergraduate entry, student flexibility, and a master’s exit qualification without increasing time.

Methods

The method included a curriculum review in 2013 where its problem-based learning curriculum shifted from a seven to a five-semester program; changing the third year to a virtual hospital clinical year using simulation, and introducing in 2016 a new 3+2 curriculum model in the final two years using a 100 point system as a masters level program.

Results

The MD model was described in the external evaluation as ‘novel and innovative’, where students can choose from three project options - a research project, or a professional project or an international capstone experience as well as a number of scholarly tasks. The structure is fully integrated with the existing curriculum and assessment process, supported by an innovative technology platform.

Conclusion

Now in its third year of implementation this innovative model is breaking new ground in the way in which a masters level MD program could be developed, whilst maintaining undergraduate entry.

## Introduction

Many medical programs internationally have moved to the US style Doctor of Medicine (MD) program. Australia is no different, with its twenty medical schools offering undergraduate or post graduate entry, a variety of 4 year, 5 year and 6 year programs that meet the Australian Qualification Framework (AQF) level 7 (Bachelor), 8 (honours) and 9 (masters) requirements, yet all achieving the same Australian Medical Council accreditation ( Cheek *et al.,* 2016). The main difference between level 7 and level 9 programs is largely research training. This paper describes one such transition that offers insights into the unique 3+2 MD model, its innovations used and challenges that other universities may learn from.

## Background

Bond University on the Gold Coast in Australia enrolled its first medical students in 2005 with a program adapted from the Sydney University 1997 Graduate Medical program. The Bond program was designed as a 4 year and 8-month program or 14 academic semesters long. The first 7 semesters used problem-based learning (PBL), the 8 ^th^ semester was preparation for the clinical rotations and the final two years were clinical rotations.

## Methodology

In 2013 Bond reviewed its curriculum, which resulted its problem-based learning curriculum shifting from a seven to a five-semester program; changing the third year to a virtual hospital clinical year using simulation. In 2016 the medical program transitioned from a MBBS to a Bachelor of Medical Studies/Doctor of Medicine Degree (MD), using an innovative 3+2 model, which allows students to enrol at undergraduate level yet graduate with a master’s degree ( Smith, 2014). These curriculum innovations provided space for the research training and project requirements that increased clinical exposure and provided flexibility for student choice. In 2016, the Australian Medical Council accredited the program for 6 years. Over 200 medical students have graduated with the BMedSt/MD since 2017.

The curriculum model was based on the following principles: it was built on what existed, sustainable, flexible, outcomes focussed, multiple exit points, fully integrated, educationally sound, and undertaken in a supportive environment that met staff capacity. An additional scholarship component was designed to overlay the existing curriculum. Specifically, students must attain 100 MD points over the final three years; 90 points are ‘core activities’ and 10 are ‘elective activities’.

The scholarship component provides students activities that structure their understanding and experience of the research process from beginning to end. The activities commence with a literature review and completion of research modules in year 3, three clerked cases and choosing their project topic at the *MD Roadshow* in year 4, through to completion of the project, submission of an abstract and a conference presentation at the *End of Program Conference* in Year 5.

The main activity (40 points) is the undertaking of a project from three options, (1) a research project e.g., an empirical study, (2) a professional project e.g., medical education project or simulation activity; or (3) a health equity capstone project e.g., undertaken at Solomon Islands, Cape York in Australia, India, or Capetown in South Africa. Students are required to undertake 100-120 hours of work on their respective project and write a 2,500-word report, as described in Table 1.

**Table 1.** MD Program Structure

Year	Core Activity	Points	Description	Total points
**3**	Critically Appraised Topic	5	Oral Presentation	5
	Two online Core Research Modules	5 each	1. Searching for a literature review 2. Developing an answerable research question	10
	Literature review	10	Written assignment - 1500 words from choice of six topics	10
***AQF LEVEL 7*** - students may exit with a Bachelor of Medical Studies				
**4**	Apply for an MD Project	Required	Three options: 1. Research Project 2. Professional Project 3. Capstone Project	Nil
	Submit a Project Plan	Required	Written and agreed Project Plan and Timeline	Nil
	3 Clerked Cases	5 each	Written 1000-word case	15
**5**	Submit MD Project Report	40	2500-word report	40
	Submit a Conference Abstract	5	250-word Abstract	5
	Present at Student Research Conference	5	Oral, group or poster presentation	5
**90 CORE POINTS**				
**Year**	**Elective Activity**	**Points**	**Description**	**Total points**
1-5	Evidence of leadership, scholarship, volunteering, or professional development activities	5 each	Extracurricular professional activities i.e. publications, conferences, committee work	5-10
3-5	Additional Critically Appraised Topic	5	Oral presentation	5
4-5	Additional Clerked Case	5	Written 1000-word case	5
4-5	Additional research modules	5 each	Students choice	5
**10 ELECTIVE POINTS**				
**Submission of final PORTFOLIO - 90 CORE POINTS AND 10 ELECTIVE POINTS - TOTAL 100 pts**				
***AQF LEVEL 9(E)*** - Students Graduate with BMedSt and MD.				

## Results

The program, which was accredited by the Bond University Senate and Australian Medical Council in 2016, is now in its third year of implementation. Of the 84 students in the first graduating MD cohort, 43 undertook a research project that was supervised by clinicians from feeder hospitals and a Bond co-supervisor, 19 undertook professional projects, and 22 undertook capstone projects during their final year. Table 2 describes the distribution of student projects from 2017-19.

**Table 2.** Distribution of student projects 2017-19

Project types	Year 5 ( *n*=96) 2019	Year 5 ( *n*=93) 2018	Year 5 ( *n*= 84) 2017
**Research projects**	43 students x 23 projects	50 students x 21 projects	43 students x16 projects
**Professional projects**	14 students x 4 projects	14 students x 3 projects	19 students x 4 projects
**Capstone projects**	39 students x 4 immersions	29 students x 3 immersions	22 students x 4 immersions

### Innovations

A number of innovations were developed during the transition process. An *electronic portfolio* was built using the university’s Blackboard technology, providing students with information, learning outcomes, project descriptions, application templates for all requirements, and used gradebook to collect and monitor assessment i.e., 100 points. The i *ntegrated structured program* ensured students followed a guided scholarly experience that was fully integrated into the existing program. Online research modules conducting a literature search were developed, and an existing assignment was adapted for assessment of writing a literature review.

*Flexibility* was a priority, given that not all students wanted to undertake research. Following AQF requirements students chose from three project types - research project, professional project, or capstone project. They were then given freedom of choice from a bank of projects across a range of medical domains e.g., oncology, mental health, obstetrics, general practice etc. These projects resulted from an expression of interest from clinicians in feeder hospitals as well as academic staff. This process was undertaken well ahead of time (6-8 months) to enable the project supervisor to gain ethics approval if required and for clinical placements to be allocated. An *MD Project Roadshow* was held at the commencement of 4 ^th^ year for clinicians and academic staff to ‘pitch’ their projects to the students, followed by discussion time with clinicians.

The c *apstone health equity immersion* option was a unique feature of the program. During the final year, students could elect to undertake a 7-week placement at one of three sites: Kira Kira Hospital (Solomon Islands), Capetown (South Africa), and Apunipima Health Council (Cape York), and in 2018 India. The placement was supported by an online Global Health Module, a clinical skills update workshop, a pre-departure briefing session and post-immersion debriefing session. Evaluation feedback has been extremely positive with many students stating that these placements have changed their future career trajectory ( Smith, Jones and Fink, 2015).

A successful *supervision* process was vital to the success of the students’ research experience. Each project was allocated a project supervisor, and external clinicians were also allocated a ‘Bond co-supervisor’, whose role was to provide a direct link to the university, support the supervisor and the students, monitor the project to ensure timelines were met, and to assist with methodological advice as required. A highlight of the program was the *End-of-Program* *Conference.* All Bond medical students, clinicians, supervisors, and academic staff attend ( *n*=approximately 500). Final year students present their project work and contribute to a published book of abstracts.

### Challenges

Several challenges need mention. The MD model is quite different to other universities, so it was important to communicate and draw out these variances. Sourcing sufficient projects, across a range of topics, was initially thought to be a challenge, however, this was not the case. Using the expression of interest process for clinical projects provided ample (four-fold) for our needs, increasing in the subsequent years. We also provided two academics for qualitative and quantitative methodological advice. The co-supervisors were also an asset for each project.

With any new initiative it was imperative to include all staff and students, during the developing stages of the program. Communication was the key to our success. We ran regular information sessions for all students, hospitals, academic and professional staff to ensure they engaged with the transition process. Our development team comprised three academics sharing the workload (Total = 1.0 FTE), and one part-time administrator. An Implementation Committee oversaw the process, comprising clinicians, hospital placement coordinators, academics, and administrators. In the final year, management of the implementation was handed to the medical program assessment team with an MD academic lead guiding the process. Working with multiple hospitals and two universities was not without complication. Sometimes the students shared projects with another university which used a different model (i.e., research experience was optional and extra-curricular), there was at times some confusion regarding the different student requirements. Problems were ultimately resolved using the co-supervisors.

## External Evaluation

The transition from MBBS to MD was externally evaluated during the 18-month transition using a case study approach. Mixed methods were used to collect documentary evidence, participant perceptions and field notes. Data collection was staged to collect executive and administrative staff perspectives of the transition; supervisor and student perspectives of the MD projects and experiences. A total of 26 interviews were conducted with university staff, students, or clinical staff who supervised the projects. An interim report was provided which applied a developmental evaluation framework ( Patton, 2011).

Staff considered the implementation had been managed very well, the product looked ‘slick’, and the flexibility of project options was a strength. The MD project was viewed as a new and novel approach that it offered student choice of experience, which aligned with a student-centred learning approach ( Cheek, 2018). The international capstone projects were in high demand and enabled over 30-40 students per annum to immerse themselves in resource-poor remote communities in small organised groups.

The scope of the available research projects was broad, with many supervised by clinicians who were novice researchers. Pairing clinical supervisors with an academic co-supervisor, achieved some good outcomes, which were directly translatable in the clinical setting. For example, one clinical audit of vaginal breech deliveries found that, so few were being performed that safety could not be presumed if the principal factor was obstetrician skill and experience. These small projects provide an opportunity for small research hubs to be established in clinical settings. While supervisor support was found to be both a strength and a weakness, and the evaluation recommended supervisor training which was offered in 2018.

The e-portfolio and online resources and communication systems contributed to the scholarship experience, in particular the two bookends of the *MD Project Roadshow* and *End of Program Conference,* which cultivated student and clinician enthusiasm and showcased the work of the graduating students ( Cheek, 2018). While the decision to transition from the MB BS did not initially have universal staff support, it was now well accepted as the new program. One excellent professional project example was undertaken by a small group of students developing an examination question bank, which they blueprinted, and quality approved under the guidance of the assessment lead. This resource is now being used by year 1 students in practice exams and is an ongoing student project.

Areas for improvement were in the variable quality of some research projects, the potential to overload busy faculty, clinical supervisors, and clinical settings; and communicating the expectations of the program between the university and the clinician. However, clinicians interviewed also acknowledged the collaborative relationships being built and that these projects were forcing them to think more critically about the care they were providing and enabling research that may not have otherwise been possible ( Cheek, 2018). The collaborative arrangements with the community health organisations were found to be paramount to the continued success of the research and capstone projects, which require shared ownership by all organisations.

## Conclusion

The transition from MBBS to an undergraduate entry MD provides an innovative platform for future cohorts. The process and evaluation may be helpful for other university’s medical programs to broaden their scholarship options for students. The strength of the process was the innovative model, flexibility, and choice for students. The collaboration and communication initiatives, the e-portfolio, the small diverse team who designed, developed, and oversaw the implementation of the program, and strong leadership from executive were essential to the success of the process.

## Take Home Messages

• Presents an innovative and novel curriculum model
• Allows students choice from three project pathways
• Allows undergraduate entry with masters exit without increasing time
• Fully integrated with existing curriculum and assessment process using 100 MD points
• Supported by an electronic MD Portfolio
• Externally evaluated

## Notes On Contributors

Janie Dade Smith is the Professor of Innovations in Medical Education at the Faculty of Health Sciences and Medicine at Bond University in Australia.

Elizabeth Edwards is Programme Director, Doctorate of Counselling Psychology, School of Psychological & Social Sciences, York St John University, UK and previously worked at Bond University.

Peter D Jones is a Professor of Paediatrics and previous Dean of Medicine at Bond University.

Colleen Cheek is a PhD student at the University of Tasmania.

Richard B Hays is Professor of Medical Education at University of Tasmania.

## Declarations

Richard Hays is the Editor of MedEdPublish. Janie Smith and Richard Hays were PhD supervisors for Colleen Cheek. No other potential conflicts are declared.
