# Supplementary material for: Transition from an MBBS to an MD program – Using innovation and thinking outside the square
Source: MedEdPublish (2016). 2019 Nov 4;8:197. [Version 1] doi: 10.15694/mep.2019.000197.1 (PMC10712541; doi:10.15694/mep.2019.000197.1)
Supplement: Peer review history

# Reviewer report: Reviewer response for version 1

**DOI:** 10.21956/mep.19911.r30166

**Published:** 2019-11-24

**Authors:** P Ravi Shankar

**Affiliations:** 1 American International Medical University

This review has been migrated. The reviewer awarded 4 stars out of 5 This is an interesting and well written manuscript. The authors describe the transition from a MBBS to a MD program at an Australian medical school. The authors have concentrated on describing the research component of the program. The research project has been well described. The process and the challenges have been highlighted. I am not sure if the authors have addressed other curricular modifications to the program elsewhere. Where there other modifications to the curriculum during the transition? Were there any changes to the duration of the program? What is meant by the new 3 + 2 curriculum model? Table 1 show that at the end of year 3 students may exit with a Bachelor of Medical Studies. Is this equivalent to MBBS? Are these graduates eligible to practice medicine? In the ethics statement section I did not understand the term ‘gatekeeper approval’. This article will be of interest to all medical educators.

Reviewer Expertise:

NA

No decision status is available

# Reviewer report: Reviewer response for version 1

**DOI:** 10.21956/mep.19911.r30164

**Published:** 2019-11-22

**Authors:** Wendy Hu

**Affiliations:** 1 School of Medicine, Western Sydney University

This review has been migrated. The reviewer awarded 4 stars out of 5 This is a clear, factual and informative account of the transition to a MD or professional masters course from the traditional bachelors level qualification, and very interesting to read from within the Australian context. Perhaps there are global lessons to be learnt about how curriculum "movements" begin and evolve in certain contexts and countries, united by affordances in a shared accreditation and higher education qualification system. Bond's leading innovation was as the first 5 year undergraduate medical program in Australia to make the transition and there may be unique insititutional levers. In contrast to the traditional research based masters, the Australian qualifications framework allows for a professional "extended" masters where the autonomous component does not necessarily require research, but any professionally focused project. Given the pressures and value that students perceive on doing "research" it is interesting to look back over three years and collate the type of projects students have chosen. It appears the proportion of non-research projects has remained similar. Has an evaluation been conducted on the reasons? Was it due to availability, allocation or true choice? Do students develop their own, or choose from a pre-defined list? Its noted that the projects are sometimes of variable quality; did this arise from different expectations for different types of projects, or other reasons? Finally after three years, has the roll out of projects and engagement with project supervisors impacted on local research and scholarly capacity? As with all thought provoking reports, more important questions are raised by the work, so thank you for sharing.

Reviewer Expertise:

NA

No decision status is available

# Reviewer report: Reviewer response for version 1

**DOI:** 10.21956/mep.19911.r30163

**Published:** 2019-11-15

**Authors:** Sateesh Babu Arja

**Affiliations:** 1 Avalon University School of Medicine

This review has been migrated. The reviewer awarded 4 stars out of 5 The authors detailed the process very well. A very well documented transition process. But I am also uncertain the reason for transition from MBBS to MD program. I am assuming that MBBS program might have optional involvement in research work and it could be an elective based on students' interest. But with MD program, students are required mandatory to be involved in research project. It is very important for the medical graduates to be familiar with the reteach methodology and be able to conduct research independently when they become future physicians and or scientists.

Reviewer Expertise:

NA

No decision status is available

# Reviewer report: Reviewer response for version 1

**DOI:** 10.21956/mep.19911.r30165

**Published:** 2019-11-05

**Authors:** Trudie Roberts

**Affiliations:** 1 Leeds Institute of Medical Education

This review has been migrated. The reviewer awarded 3 stars out of 5 An interesting descriptive account of innovation in a school's curriculum. I am uncertain why the School wanted to move to an MD programme. Why was this thought to be better. The project is interesting but many schools around the world have significant projects within their programme without moving to an MD programme

Reviewer Expertise:

NA

No decision status is available
